# Inflammation Induced by Lipopolysaccharide and Palmitic Acid Increases Cholesterol Accumulation via Enhancing Myeloid Differentiation Factor 88 Expression in HepG2 Cells

**DOI:** 10.3390/ph15070813

**Published:** 2022-06-30

**Authors:** Junbin Chen, Yuguo Liu, Huiyu Luo, Guoxun Chen, Zhongdaixi Zheng, Tiannan Wang, Xinge Hu, Yue Zhao, Jiaqi Tang, Chuhong Su, Longying Zha

**Affiliations:** 1 Department of Nutrition and Food Hygiene, Guangdong Provincial Key Laboratory of Tropical Disease Research, School of Public Health, Southern Medical University, Guangzhou 510515, China; box@i.smu.edu.cn (J.C.); Liuyuguo12300@163.com (Y.L.); luohuiyu0414smu@163.com (H.L.); kangaroodaixi927@163.com (Z.Z.); lunazhao99@163.com (Y.Z.); jiaqitang.fjly@foxmail.com (J.T.); chuhongsu@163.com (C.S.); 2 Department of Nutrition, University of Tennessee at Knoxville, Knoxville, TN 37996, USA; gchen6@utk.edu (G.C.); twang14@vols.utk.edu (T.W.); xhu25@vols.utk.edu (X.H.)

**Keywords:** lipopolysaccharide, palmitic acid, toll-like receptor 4, myeloid differentiation factor 88, sterol regulatory element-binding protein-2, inflammation, cholesterol accumulation

## Abstract

Recently, multiple studies have shown that chronic inflammation disturbs cholesterol homeostasis and promotes its accumulation in the liver. The underlying molecular mechanism remains to be revealed. The relationship between the toll-like receptor 4 (TLR4) inflammatory signaling pathway and cholesterol accumulation was investigated in HepG2 cells treated with lipopolysaccharide (LPS) or palmitic acid (PA) for different lengths of time. In addition, the effects of pretreatment with 20μmol/L ST2825 (MyD88 inhibitor) were also studied in LPS- or PA-treated HepG2 cells and myeloid differentiation factor 88 (MyD88)-overexpressing HEK293T cells. The intracellular total and free cholesterol levels were measured using a commercial kit and filipin staining, respectively. The expression levels of sterol regulatory element-binding protein-2 (SREBP-2) and components in the TLR4 signaling pathway were determined using Western blotting. The treatments with LPS for 12 h and with PA for 24 h significantly increased the contents of intracellular total and free cholesterol, as well as the expression levels of SREBP-2 and components in the TLR4 signaling pathway. The inhibition of MyD88 by ST2825 significantly decreased the cholesterol content and the expression levels of SREBP-2 and components of the TLR4/MyD88/NF-κB pathway in HepG2 cells, as well as MyD88-overexpressing HEK293T cells. These results indicated that LPS and PA treatments increase SREBP-2-mediated cholesterol accumulation via the activation of the TLR4/MyD88/NF-κB signaling pathway in HepG2 cells.

## 1. Introduction

Chronic inflammation is strongly associated with a series of chronic metabolic diseases, such as diabetes, obesity, cardiovascular diseases, nonalcoholic fatty liver diseases, etc. [1,2]. It promotes the dysregulation of lipid metabolisms [3,4], especially the cholesterol homeostasis characterized by cholesterol accumulation in hepatocytes [5,6]. Excessive cholesterol accumulation affects the functions of hepatocytes and immune cells, which leads to the development of chronic metabolic diseases [7]. Lipopolysaccharide (LPS) is a glycolipid found in the cell wall of Gram-negative bacteria, and palmitic acid (PA) is 16-carbon saturated fatty acid rich in high-fat diets (HFDs). Prolonged exposure of low levels of LPS and PA results in chronic inflammation and cholesterol accumulation [8]. It is known that the feeding of a HFD impairs the enteric epithelial barrier and increases intestinal permeability [9,10], which led to an increase in circulating LPS levels and inflammation in the liver and adipose tissues of C57BL/6J mice [9]. Many studies have shown that LPS and PA can interact with toll-like receptor 4 (TLR4) [11,12].

The TLR4 signaling pathway is an important part of chronic inflammation [13]. Upon activation, TLR4 can trigger MyD88-dependent and TIR-domain-containing adaptor-inducing interferon (IFN)-β (TRIF)-dependent signaling pathways in hepatocytes and immune cells [14,15]. In the MyD88-dependent pathway, MyD88 is recruited by TLR4 and activates interleukin-1 receptor-associated kinases (IRAKs) and tumor necrosis factor receptor-associated factor 6 (TRAF6), leading to the early-phase activation of nuclear factor-κB (NF-κB). In the TRIF-dependent pathway, TLR4 is endocytosed in intracellular vesicles and forms a complex with TRIF, which regulates the expression of type I IFN by TRAF3 [16]. The TRIF-dependent pathway also can regulate the late-phase activation of NF-κB by TRAF6 [17]. Both signaling pathways of TLR4 can stimulate the production of pro-inflammatory cytokines, such as TNF-α, IL-1β, and IL-6 [16]. Recent studies have indicated that the activation of the TLR4 signaling pathway is associated with intracellular cholesterol accumulation, leading to the disturbance of cholesterol homeostasis in hepatic cells [5,18].

The liver plays an important role in maintaining cholesterol homeostasis, which is regulated by enzymes and transcription factors involved in hepatic cholesterol biosynthesis and utilization [19]. One key transcription factor for cholesterol biosynthesis is sterol regulatory element-binding protein 2 (SREBP-2). When the intracellular cholesterol level falls, SREBP-2 is transferred from the endoplasmic reticulum to the Golgi after being processed for maturation. The mature form of SREBP-2 (N-SREBP-2) enters the nucleus and induces the expression levels of genes involved in cholesterol homeostasis, including LDL-receptor (LDLR) and 3-hydroxy-3-methylglutaryl CoA reductase (HMG-CoAR), which control the cholesterol uptake and synthesis, respectively [20]. In addition, SREBP-2 also indirectly affects the expression of ABCA1 and ABCG1, which mediate cholesterol efflux, by co-expressing microRNA-33a [21]. Recent studies have indicated that IL-1β, TNF-α, and other cytokines perturb cholesterol homeostasis by increasing SREBP-2-mediated cholesterol synthesis in macrophages and hepatocytes [18,22].

MyD88 is a key mediator in the TLR4 inflammatory signaling pathway and may contribute to LPS- and PA-activated cholesterol accumulation in hepatic cells. It acts as a nutrient sensor and controls energy expenditure in intestinal epithelial cells [23]. Additionally, some phytochemicals (syringic acid and resveratrol) could reduce the expression of MyD88 and SREBP-2 in the liver and adipose tissues of HFD-fed mice [24,25,26]. These studies have indicated that MyD88 may modulate cholesterol accumulation by regulating the SREBP-2 expression.

However, the role of MyD88 in LPS- and PA-activated hepatic cholesterol accumulation and the underlying mechanisms remain unclear. We hypothesize that activation of the TLR4 signaling pathway induced by LPS and PA increases SREBP-2-mediated cholesterol synthesis via enhancing MyD88 expression. Here, this hypothesis is tested in HepG2 cells treated with or without LPS or PA.

## 2. Results

### 2.1. LPS and PA Treatments Inhibited HepG2 Cell Viabilities

**Figure 1:**
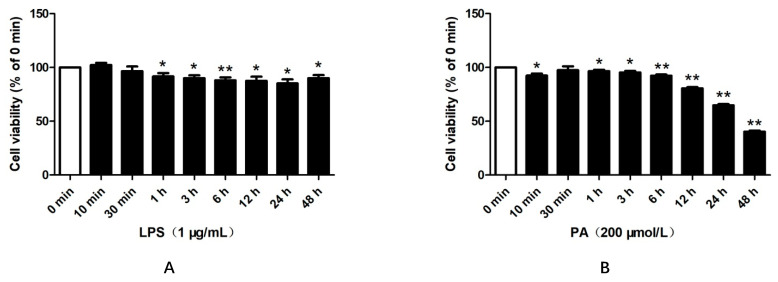
Effects of LPS and PA on the viabilities of HepG2 cells. Cells were treated with 1 μg/mL LPS (**A**) or 200 μmol/L PA (**B**) for different times (0 min, 10 min, 30 min, 1 h, 3 h, 6 h, 12 h, 24 h, and 48 h). MTT assays were performed to determine the cell viability. The results are presented as means ± SD in triplicate (*n* = 6); *: *p* < 0.05 vs. 0 min; **: *p* < 0.01 vs. 0 min.

To investigate the effects of LPS and PA on HepG2 cell viabilities, the cells were treated with 1 μg/mL of LPS or 200 μmol/L of PA. Compared with that at 0 min, LPS treatments for 1 h, 3 h, 6 h, 12 h, 24 h, and 48 h (Figure 1A) or PA treatments for 10 min, 1 h, 3 h, 6 h, 12 h, 24 h, and 48 h (Figure 1B) significantly reduced the cell viabilities.

### 2.2. LPS and PA Treatments Increased Cholesterol Content in HepG2 Cells

**Figure 2:**
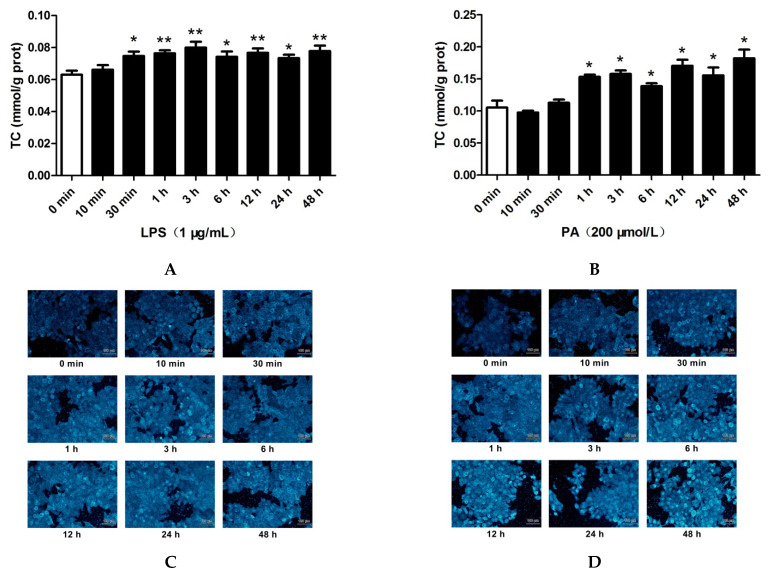

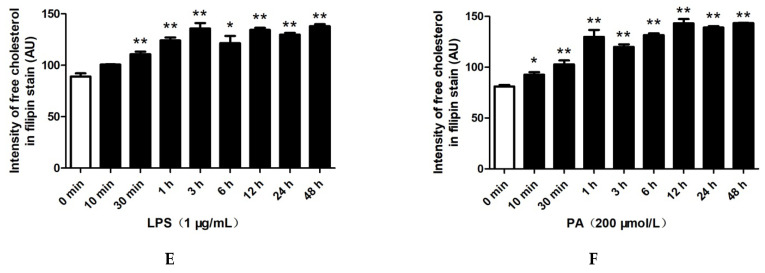
Effects of LPS and PA on cholesterol content in HepG2 cells. Cells were treated with LPS (1 μg/mL) or PA (200 μmol/L) for different times (0 min, 10 min, 30 min, 1 h, 3 h, 6 h, 12 h, 24 h, and 48 h). Intracellular total cholesterol (TC) was determined with a commercial kit (**A**,**B**). Intracellular free cholesterol was visualized by filipin staining (**C**,**D**) and quantified using ImageJ and SPSS20.0 software (**E**,**F**). The results are presented as means ± SD of three independent experiments; *: *p* < 0.05 vs. 0 min, **; *p* < 0.01 vs. 0 min.

As shown in Figure 2A,B, LPS treatment for 30 min to 48 h and PA treatments for 1 h to 48 h significantly increased the total cholesterol (TC) levels in HepG2 cells. Filipin staining was used to determine the intracellular free cholesterol levels (Figure 2C,D). The free cholesterol contents in HepG2 cells treated with LPS for 30 min to 48 h or with PA for 10 min to 48 h were significantly increased compared with that for 0 min (Figure 2E,F). These results showed that LPS and PA treatments increased the cholesterol accumulation in HepG2 cells.

### 2.3. LPS and PA Treatments Increased TLR4/MyD88/NF-κB Signaling Pathway Activation and SREBP-2 Expression

**Figure 3:**
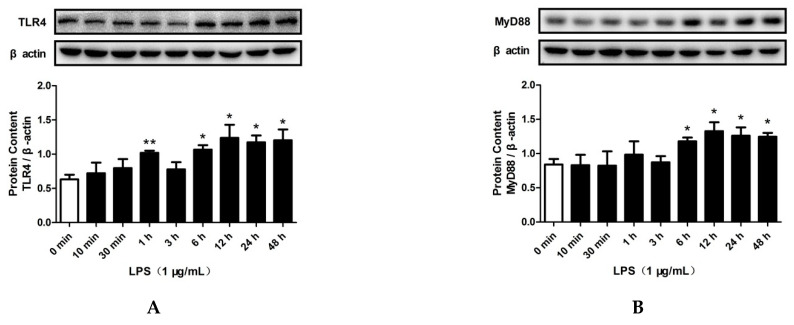

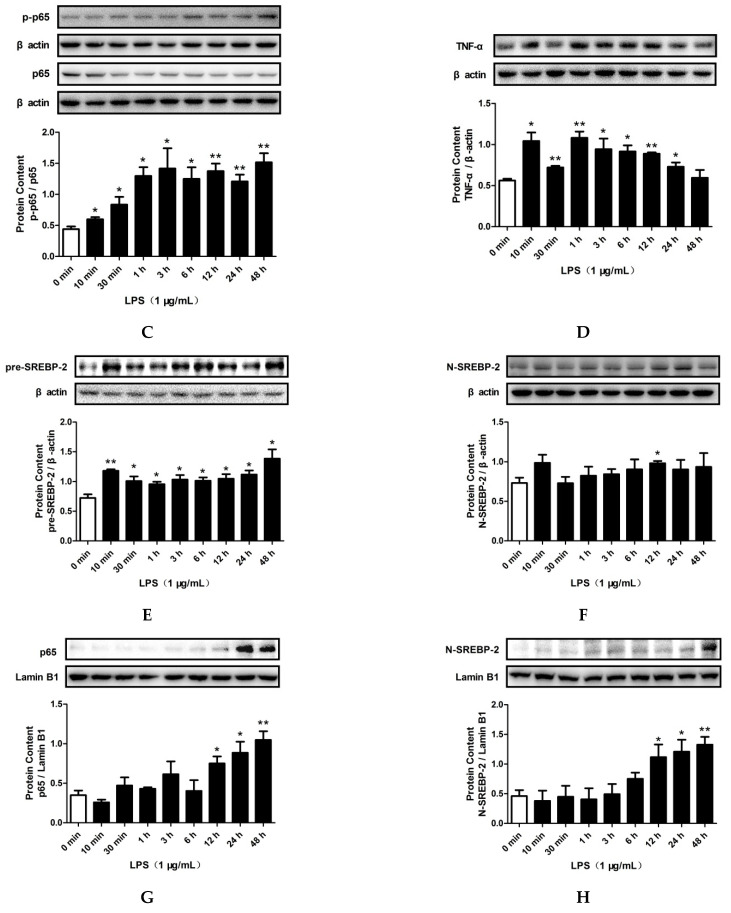
Effects of LPS on TLR4/MyD88/NF-κB signaling pathway activation and SREBP-2 expression in HepG2 cells. Cells were treated with LPS (1 μg/mL) for 0 min, 10 min, 30 min, 1 h, 3 h, 6 h, 12 h, 24 h, or 48 h. The total protein levels of TLR4 (**A**), MyD88 (**B**), TNF-α (**D**), SREBP-2 precursor (pre-SREBP-2) (**E**), SREBP-2 mature forms (N-SREBP-2) (**F**), and p65 phosphorylation (**C**) were measured by Western blotting. The nuclear protein levels of p65 (**G**) and N-SREBP-2 (**H**) were detected by Western blotting. The results are presented as means ± SD of three independent experiments; *: *p* < 0.05 vs. 0 min; **: *p* < 0.01 vs. 0 min.

To determine the effects of LPS and PA on cholesterol accumulation induced by SREBP-2 and inflammation caused by the TLR4/MyD88/NF-κB signaling pathway, HepG2 cells were treated with LPS or PA for 0 min, 10 min, 30 min, 1 h, 3 h, 6 h, 12 h, 24 h, and 48 h. After that, the expression levels of SREBP-2 and key proteins of the TLR4/MyD88/NF-κB signaling pathway were determined. As shown in Figure S1A, LPS treatment for 10 min to 48 h did not change the protein levels of TRIF. However, LPS treatment significantly increased the protein levels of TLR4 (1 h and 6 h to 48 h), MyD88 (6 h to 48 h), TNF-α (10 min to 24 h), SREBP-2 precursor (pre-SREBP-2; 10 min to 48 h), SREBP-2 mature forms (N-SREBP-2; 12 h), and TRAF6 (1 h to 48 h), as well as NF-κB p65 phosphorylation (10 min to 48 h), as shown in Figure 3A–F and Figure S1B, respectively. Moreover, LPS treatment enhanced the nuclear translocation of the NF-κB p65 subunit (Figure 3G) and N-SREBP-2 (Figure 3H) at 12 h, 24 h, and 48 h.

**Figure 4:**
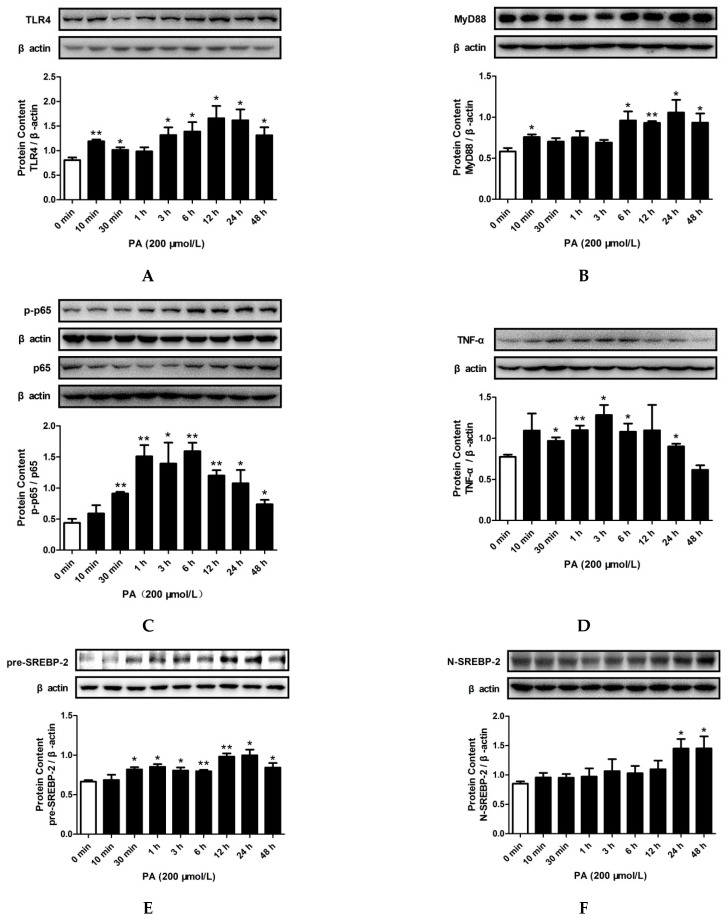

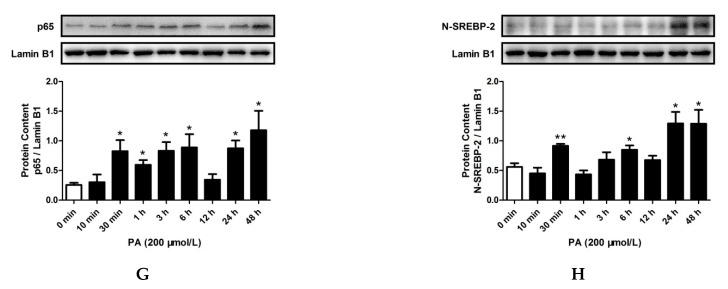
Effects of PA on TLR4/MyD88/NF-κB signaling pathway activation and SREBP-2 expression in HepG2 cells. Cells were treated with PA (200 μmol/L) for 0 min, 10 min, 30 min, 1 h, 3 h, 6 h, 12 h, 24 h, or 48 h. The total protein levels of TLR4 (**A**), MyD88 (**B**), TNF-α (**D**), pre-SREBP-2 (**E**), N-SREBP-2 (**F**), and p65 phosphorylation (**C**) were measured by Western blotting. The nuclear protein levels of p65 (**G**) and N-SREBP-2 (**H**) were detected by Western blotting. The results are presented as means ± SD of three independent experiments; *: *p* < 0.05 vs. 0 min; **: *p* < 0.01 vs. 0 min.

In addition, Figure S1C shows that PA treatment for 10 min to 48 h did not significantly alter the protein level of TRIF. However, PA treatment significantly increased the protein levels of TLR4 (10 min, 30 min, and 3 h to 48 h), MyD88 (10 min and 6 h to 48 h), TNF-α (30 min to 6 h and 24 h), pre-SREBP-2 (30 min to 48 h), N-SREBP-2 (24 h and 48 h), and TRAF6 (6 h to 48 h), as well as NF-κB p65 phosphorylation (30 min to 48 h), as shown in Figure 4A–F and Figure S1D, respectively. Furthermore, PA treatment significantly increased the nuclear levels of the NF-κB p65 subunit (30 min to 6 h, 24h, and 48 h) (Figure 4G) and N-SREBP-2 (30 min, 6 h, 24 h, and 48h) (Figure 4H).

Together, these results indicated that LPS treatment for 12 h and PA treatment for 24 h could activate the TLR4/MyD88/NF-κB signaling pathway and induce SREBP-2 expression. Therefore, 12 h and 24 h were respectively chosen as the times for LPS and PA treatments in the following experiments.

### 2.4. Inhibition of MyD88 Reduced Cholesterol Content in LPS- and PA-Treated HepG2 Cells

**Figure 5:**
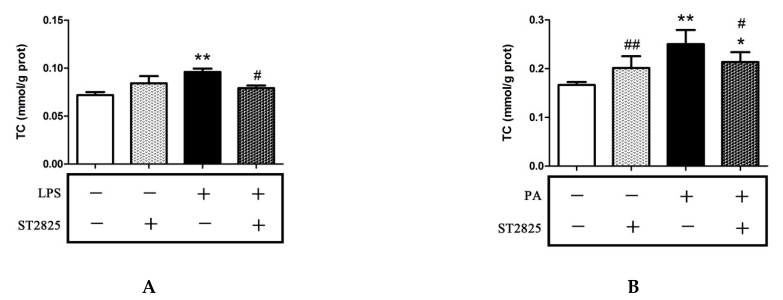

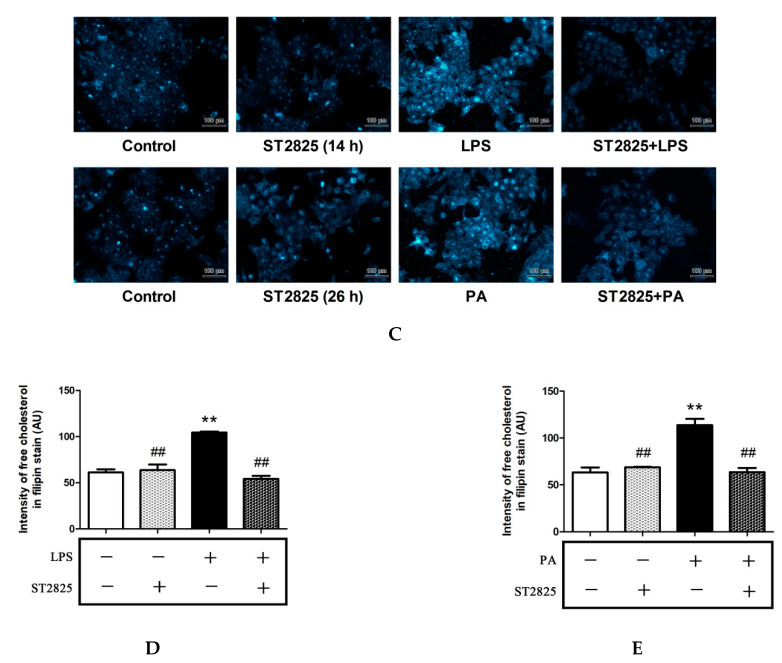
MyD88 inhibitor ST2825 reduced the cholesterol content in LPS- or PA-treated HepG2 cells. HepG2 cells were pre-treated with 20 μmol/L ST2825 for 2 h and then stimulated with LPS (1 μg/mL) for 12 h or with PA (200 μmol/L) for 24 h. Intracellular TC was determined with a commercial kit (**A**,**B**). Intracellular free cholesterol was visualized using filipin staining (**C**) and quantified with ImageJ and SPSS20.0 software (**D**,**E**). The results are presented as means ± SD of three independent experiments; *: *p* < 0.05 vs. control; **: *p* < 0.01 vs. control; #: *p* < 0.05 vs. LPS or PA alone; ##: *p* < 0.01 vs. LPS or PA alone.

MyD88 is an upstream molecule of the TLR4/MyD88/NF-κB signaling pathway that triggers the inflammation induced by LPS or PA [12,27,28]. It is known that inflammation increases the cholesterol accumulation in hepatic cells. To investigate the effect of MyD88 on cholesterol accumulation, HepG2 cells were pre-incubated with 20 μmol/L of ST2825 for 2 h and stimulated with LPS for 12 h or with PA for 24 h, and the intracellular cholesterol content was determined. Filipin was used to determine the free cholesterol in HepG2 cells [29]. As shown in Figure 5, ST2825-only treatment for 16 h and 26 h did not alter the levels of TC and free cholesterol in the HepG2 cells. LPS or PA treatment significantly increased the levels of TC and free cholesterol. This is in agreement with the results of time-dependent experiments. Compared with the LPS and PA groups, ST2825 pre-incubation significantly decreased the levels of TC and free cholesterol.

### 2.5. Inhibition of MyD88 Suppressed TLR4/MyD88/NF-κB Signaling Pathway Activation and SREBP-2 Expression Induced by LPS and PA Treatments

**Figure 6:**
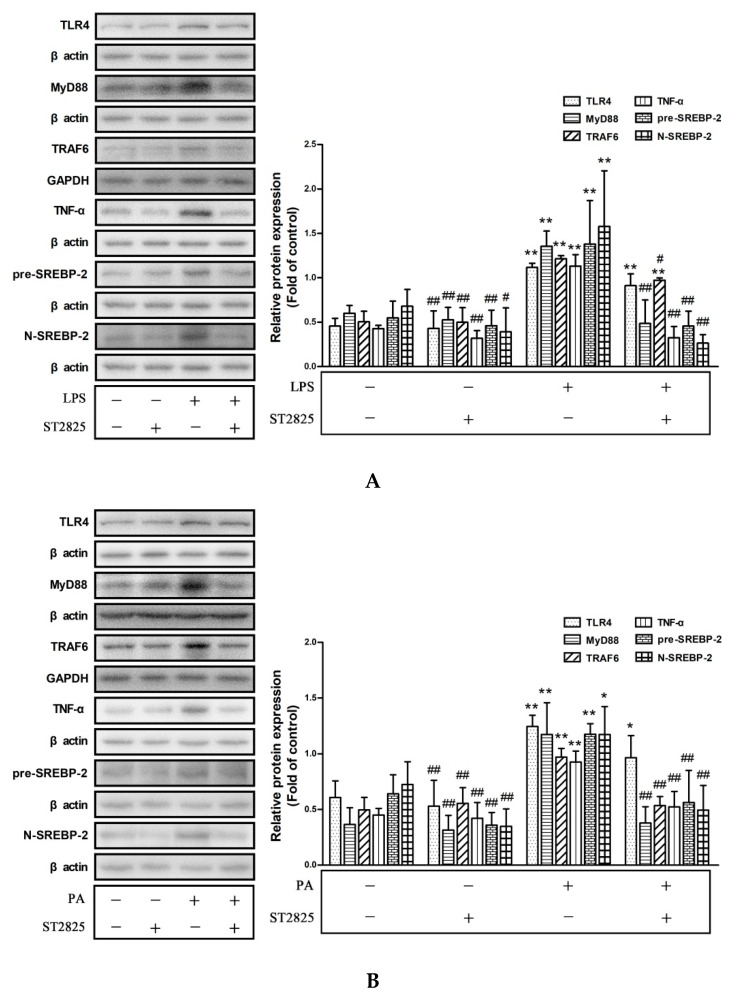
MyD88 inhibitor ST2825 suppressed inflammation and SREBP-2 expression in LPS- or PA-treated HepG2 cells. HepG2 cells were pre-treated with 20 μmol/L ST2825 for 2 h and then treated with LPS (1 μg/mL) for 12 h (**A**) or with PA (200 μmol/L) for 24 h (**B**). The protein levels of TLR4, MyD88, TRAF6, TNF-α, SREBP-2 precursor (pre-SREBP-2), and SREBP-2 mature forms (N-SREBP-2) were measured by Western blotting. The results are presented as means ± SD of three independent experiments; *: *p* < 0.05 vs. control; **: *p* < 0.01 vs. control; #: *p* < 0.05 vs. LPS or PA alone; ##: *p* < 0.01 vs. LPS or PA alone.

As shown in Figure 6A,B and Figure S2A,B, incubation with ST2825 alone for 14 h or 26 h did not change the protein levels of TLR4, MyD88, TRAF6, TNF-α, pre-SREBP-2, and N-SREBP-2, as well as NF-κB p65 phosphorylation. ST2825 pre-incubation markedly decreased the protein levels of MyD88, TRAF6, TNF-α, pre-SREBP-2, and N-SREBP-2, as well as NF-κB p65 phosphorylation, which were all significantly upregulated by LPS or PA treatment. However, the expression of TLR4 did not alter due to ST2825 pre-incubation compared with those in the LPS or PA treatment groups. These results showed that ST2825-mediated MyD88 inhibition could suppress the activation of the TLR4/MyD88/NF-κB signaling pathway and expression of SREBP-2 in LPS- and PA-treated HepG2 cells. In addition, these results demonstrated that the inflammation activated by the TLR4/MyD88/NF-κB signaling pathway promoted cholesterol accumulation via increasing the SREBP-2 expression in HepG2 cells.

### 2.6. Role of MyD88 in SREBP-2 Mediated Cholesterol Synthesis

**Figure 7:**
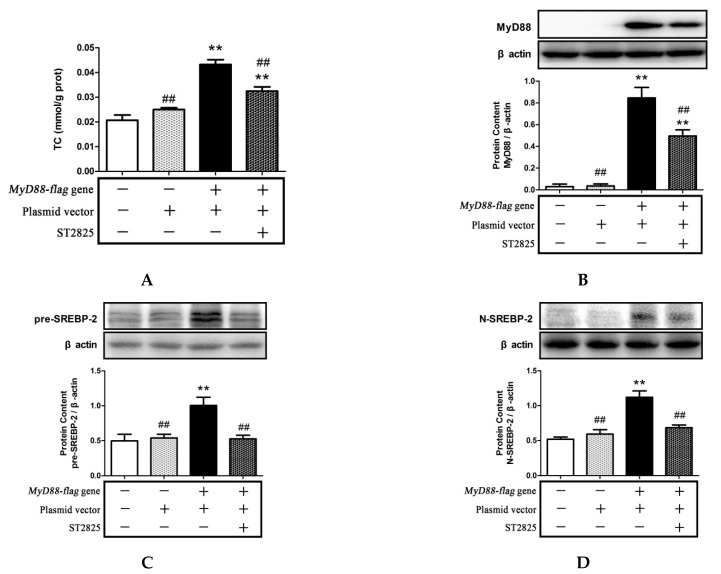
MyD88 inhibitor ST2825 inhibited cholesterol content and SREBP-2 expression in MyD88-transfected HEK293T cells. HEK293T cells were transfected with MyD88-flag plasmid for 24 h and then treated with 20 μmol/L ST2825 for 6h. Intracellular TC was determined with a commercial kit (**A**). The protein expressions of MyD88 (**B**), SREBP-2 precursor (pre-SREBP-2) (**C**), and SREBP-2 mature forms (N-SREBP-2) (**D**) were measured by Western blotting. The results are presented as means ± SD of three independent experiments; **: *p* < 0.01 vs. control; ##: *p* < 0.01 vs. MyD88-flag-plasmid-transfected group.

To further examine whether MyD88 mediates cholesterol accumulation through SREBP-2, we measured the intracellular TC level and SREBP-2 expression in HEK293T cells overexpressing MyD88 that were pre-treated with 20 μmol/L of ST2825 for 6 h. The overexpression of MyD88 was confirmed by Western blotting (Figure 7B). As shown in Figure 7, MyD88 overexpression significantly increased the intracellular TC and the protein levels of pre-SREBP-2 and N-SREBP-2. However, the inhibition of MyD88 by ST2825 significantly decreased the intracellular TC and the protein levels of pre-SREBP-2 and N-SREBP-2 compared with those in the MyD88 overexpression group. These findings indicated that MyD88 was indeed functional in the LPS- and PA- treated cells and led to cholesterol accumulation, which probably was mediated via the regulation of SREBP-2 expression.

## 3. Discussion

Chronic inflammation plays an important role in the disturbance of cholesterol homeostasis [6,18]. We and others have recently discovered that LPS, PA, and HFD can exacerbate inflammation and increase cholesterol concentration in hepatocytes and mouse livers [30,31,32]. However, the relationship between inflammation and cholesterol accumulation is not completely understood. In this study, we demonstrated that both LPS and PA could activate the TLR4/MyD88/NF-κB signaling pathway and significantly increase the intracellular cholesterol concentration in HepG2 cells. Additionally, the inhibition of MyD88 activity significantly suppressed the cholesterol accumulation mediated by SREBP-2 in LPS- and PA-treated HepG2 cells and MyD88-overexpressing HEK293T cells. These data suggested that MyD88 plays a key role in promoting the cholesterol accumulation induced by LPS and PA.

Firstly, we investigated the effects of LPS and PA on inflammation activation and cholesterol accumulation. In this study, HepG2 cells were used as a research model of inflammation activation and cholesterol accumulation induced by LPS and PA, which retain many characteristics of normal, differentiated, quiescent hepatocytes [33]. HepG2 cells are commonly used for studying the regulation of lipid metabolism [27]. Some studies have demonstrated that LPS and PA treatments can activate inflammation and cholesterol accumulation in HepG2 cells [5,34,35]. Increased plasma levels of LPS and PA have been reported in some patients with chronic metabolic diseases due to dietary and other reasons [36,37]. It has been shown that LPS treatment or HFD feeding can cause inflammation and increased cholesterol concentration in mouse livers [30,31]. In recent years, many studies also have demonstrated that treatment with low levels of LPS or PA for a long period of time can activate the TLR4 inflammatory signaling pathway and lead to chronic inflammation [15,38], which promotes cholesterol accumulation in L02 cells, Huh7 cells, primary hepatocytes, and THP-1 macrophages [5,39,40,41,42]. Similar results were obtained in this study. Interestingly, LPS and PA treatments led to a significantly increase in the activation of the TLR4/MyD88/NF-κB signaling pathway, while they did not affect the expression of TRIF in HepG2 cells, suggesting that LPS treatment for 12 h and PA treatment for 24 h only activated the TLR4 signaling system via the MyD88-depengdent pathway, but not the TRIF-dependent pathway, leading to the activation of NF-κB and the production of TNF-α. In addition, we found that LPS and PA treatments increased intracellular cholesterol levels at 30 min and 10 min. These results suggested that LPS and PA probably affect cholesterol synthesis and secretion rapidly. However, only a few studies have investigated the mechanism by which short-term treatments with LPS and PA have increased intracellular cholesterol levels. Ya-Ru et al. demonstrated that LPS treatment for 24 h significantly increased the HMGCR expression in HepG2 cells, which adjusts cholesterol synthesis [43]. PA treatment for 12 h also affected cholesterol synthesis and cholesterol secretion by the upregulation of the *FDPS* and *ABCG1* genes in HepG2 cells [44]. It was demonstrated that FDPS, ABCG1, and HMGCR expression are regulated by SREBP-2 [45].

SREBP-2 acts as a transcription factor to regulate cholesterol homeostasis in cells [46,47,48]. It was reported that inflammation induced by TNF-α and IL-1β could increase the SREBP-2 mRNA expression and intracellular cholesterol levels in primary hepatocytes [22]. Moreover, Mingyan et al. demonstrated that LPS treatment significantly increased the intracellular cholesterol content by upregulating the SREBP-2 expressions in HepG2 cells and Huh7 cells [5]. LPS increased the SREBP2 expression in HepG2 cells in a dose-dependent manner [43]. PA treatment also has upregulated cholesterol biosynthesis by increasing the SREBP-2 protein levels in HepG2 cells and L02 cells [27,39,49]. In the present study, both LPS treatment for 12 h and PA treatment for 24 h increased the levels of pre-SREBP-2 and N-SREBP-2. LPS and PA treatments also resulted in the accumulation of TC and free cholesterol in HepG2 cells. Interestingly, the trend of increased N-SREBP-2 expression over time was similar to that of NF-κB p65 in both LPS and PA treated cells. These results suggested that SREBP-2-mediated cholesterol accumulation may be associated with the inflammation activated by the TLR4/MyD88/NF-κB signaling pathway.

Secondly, we investigated the mechanism of MyD88 in SREBP-2-mediated cholesterol accumulation in HepG2 cells. MyD88 not only regulates the innate immune system, which is the adaptor protein of TLR4, IL1R, and other receptors, but it also controls the synthesis of bioactive lipids [50,51,52]. Some studies have found that LPS and PA treatments increased the MyD88 and SREBP-2 expressions in hepatocytes and macrophages [53,54]. Recently, multiple studies have focused on the role of the NF-κB signaling pathway in cholesterol accumulation, which is downstream of MyD88. It was reported that the knockdown of IKKα decreased the SREBP-2 expression and cholesterol concentration induced by LPS treatment in HepG2 cells [5]. Guojun et al. found that NF-κB specific inhibitor suppressed LPS-induced SREBP-2 expression and upregulated cholesterol efflux in apoE^-/-^ mice [21]. Nevertheless, the mechanism by which MyD88 is involved in cholesterol accumulation is not completely understood. Lung-Chih et al. indicated that the knockdown of MyD88 using siRNA inhibited SREBP-2-mediated cholesterol synthesis and uptake after LPS or IL-1β treatment [18]. However, the effect of MyD88 on SREBP-2-mediated cholesterol accumulation under PA treatment has not been investigated until now.

In this study, the ST2825-mediated inhibition of MyD88 significantly reduced the LPS- or PA-activated TLR4/MyD88/NF-κB signaling pathway, SREBP-2 expression, free cholesterol, and TC concentration in HepG2 cells. Our results, as well as others, indicated that the LPS- or PA-activated TLR4/MyD88/NF-κB signaling pathway promotes SREBP-2-mediated cholesterol accumulation. Moreover, we also showed that MyD88 can regulate cholesterol content by the SREBP-2 expression in hepatocytes after PA treatment. In order to exclude the influence of other factors induced by LPS and PA, we treated HEK293T cells overexpressing MyD88 with ST2825 to further investigate its role in cholesterol accumulation. Our previous work showed that MyD88 overexpression could activate inflammation in HEK293T cells, which normally express low MyD88, and ST2825 had an anti-inflammatory ability through suppressing MyD88 activity in MyD88-overexpressing HEK293T cells [28]. In the present study, MyD88 overexpression increased the SREBP-2 expression (pre-SREBP-2 and N-SREBP-2) and TC concentration in the cells, while this effect could be attenuated in the presence of ST2825. Together, these results suggest that MyD88 is probably the key mediator in cholesterol accumulation by regulating SREBP-2 expression.

In summary, we experimentally demonstrated that the LPS- and PA-activated TLR4/MyD88/NF-κB signaling pathway triggered an increase in intracellular cholesterol levels by promoting the expression of SREBP-2 in HepG2 cells. The major limitation of the present study was that the effects of the LPS and PA treatments on the TLR4 inflammatory signaling pathway and cholesterol accumulation were only investigated in HepG2 cells. However, we preliminarily investigated the mechanism by which MyD88 was involved in cholesterol accumulation. MyD88 was not only a regulator of inflammation, but also a meditator of SREBP-2-induced cholesterol accumulation. This broadened the understanding of the mechanisms by which LPS- and PA-activated chronic inflammation disturbs cholesterol homeostasis and implied that MyD88 inhibition may help to prevent LPS- and PA-activated cholesterol accumulation in hepatocytes.

## 4. Materials and Methods

### 4.1. Reagents and Chemicals

PA, LPS, dimethyl sulfoxide (DMSO), fatty-acid-free bovine serum albumin (BSA), filipin complex, and 3- (4, 5-dimethylthiazol-2-yl)-2, 5-diphenyl-tetrazolium bromide (MTT) were purchased from Sigma-Aldrich (St. Louis, MO, USA). ST2825 was obtained from MedChemExpress (Monmouth Junction, NJ, USA). The primary antibodies are listed in Table 1. The antibody to TLR4 (25) was purchased from Santa Cruz Biotechnology, Inc. (Santa Cruz, CA, USA). The antibody for GAPDH was purchased from Good Here (Hangzhou, Zhejiang, China). Antibodies against TRIF and TRAF6 were purchased from Abcam (Cambridge, MA, USA). Antibodies for SREBP-2 and Lamin B1 were purchased from Proteintech Group (Chicago, IL, USA). The antibodies for MyD88, TNF-α, NF-κB-p65, phospho-NF-κB-p65, β-actin, anti-rabbit IgG (1:5000; cat. no. 7074S), and anti-mouse IgG (1:5000; cat. no. 7076S) used for Western blotting were purchased from Cell Signaling Technology, Inc. (Danvers, MA, USA).

### 4.2. Cell Culture

HepG2 cells (ATCC^®^ HB-8065™) and HEK293T cells (ATCC^®^ CRL-11268™) were cultured in DMEM (Gibco, Grand Island, NY, USA) containing 10% (*v*/*v*) fetal bovine serum (Clark, Richmond, VA, USA) and 1% (*v*/*v*) penicillin-streptomycin in a humidified atmosphere containing 5% CO2 at 37 °C. HepG2 cells have been used to understand the regulation of cholesterol metabolism [27]. The cells were cultured to 80% confluence and then treated with LPS or PA in serum-free medium (DMEM containing 1% (*v*/*v*) penicillin-streptomycin).

Fatty-acid-free BSA was used to prepare the PA solution. In brief, 10 mmol/L solution of PA in 10 mmol/L NaOH was incubated at 70 °C for 30 min. Then, 10 mmol/L PA was added to equal an amount of 30% fatty-acid-free BSA at 55 °C, vortexed for 30 s, and further incubated for 15 min at 55 °C. For the LPS and PA studies, HepG2 cells were treated with LPS (1 μg/mL) or PA (200 μmol/L) for 0 min, 10 min, 30 min, 1 h, 3 h, 6 h, 12 h, 24 h, and 48 h. For the MyD88 inhibitor ST2825 study, HepG2 cells were pre-incubated with 20 μmol/L ST2825 for 2 h and then treated with 1 μg/mL LPS or 200 μmol/L PA for suitable times, as indicated in the Figure legends.

### 4.3. MTT Cell Viability Assay

An MTT assay was performed to evaluate the effects of LPS and PA on the viabilities of HepG2 cells. An amount of 1 × 10^4^/well HepG2 cells were seeded into 96-well plates 24 h before treatment. LPS or PA was then added, and the cells were incubated for 0 min, 10 min, 30 min, 1 h, 3 h, 6 h, 12 h, 24 h, and 48 h. Twenty microliters of MTT solution (5 mg/mL in PBS) was add to each well and cultured for an additional 4 h. The culture supernatants were subsequently removed, and 150 μL DMSO was supplemented in each well to dissolve the black-blue crystals. The absorbance at 570 nm was measured using a BioTek Elx808 microplate reader (Winooski, VT, USA).

### 4.4. Transient Transfection

Transient transfection was performed as described previously [28]. In brief, 1 × 10^6^/well HEK293T cells were seeded into 6-well plates and incubated overnight. The cells were transfected with 2.5 μg MyD88-flag plasmid DNA (Addgene plasmid #13093) per well using lipofectamine 3000 (Invitrogen, Carlsbad, CA, USA), according to the manufacturer’s instructions. HEK293T cells were transfected for 24 h and then treated with 20 μmol/L of ST2825 for 6 h.

### 4.5. Measurement of Intracellular Cholesterol

The cells samples were homogenized in RIPA lysis buffer (KeyGEN Biotech, Nanjing, Jiangsu, China). The concentration of total cholesterol (TC) in the cell lysis was determined using a TC kit purchased from the Nanjing Jiancheng Institute of Biotechnology (Nanjing, Jiangsu, China) with the recommended procedures.

### 4.6. Filipin Staining

Filipin is a profluorescent compound that binds to free cholesterol [29]. Briefly, HepG2 cells were grown overnight on coverslips. After different treatments, the cells were washed once with PBS, fixed with 4% paraformaldehyde, and incubated with 50 μg/mL filipin in PBS with 10% FBS for 2 h at room temperature. Fluorescence signals were captured with an Olympus IX73 fluorescence microscope (Tokyo, Japan).

### 4.7. Western Blotting

The total protein of cells lysed in RIPA lysis buffer and the nuclear protein were extracted using a nuclear extraction kit (KeyGEN Biotech, Nanjing, Jiangsu, China) containing 1mM PMSF, 1% protease inhibitors, and 0.8% phosphatase inhibitors. The samples were separated on 10% sodium dodecyl sulfate polyacrylamide gels through electrophoresis. The separated proteins were transferred onto PVDF membranes (Millipore, Billerica, MA, USA), which were blocked with 5% BSA in Tris-buffered saline buffer with Tween-20 (TBST) for 2 h at room temperature. The presence of proteins was detected through immunoblotting with primary antibodies overnight at 4 °C, followed by HRP-conjugated secondary IgG antibodies. All protein bands were developed using enhanced chemiluminescence and visualized with a Tanon-5200 imaging system (Shanghai, China).

### 4.8. Statistical Analysis

The results were analyzed with Student’s *t*-test or one-way analysis of variance (ANOVA), followed by LSD or Dunnett’s T3 multiple comparison tests using SPSS 20.0 statistical software (Chicago, IL, USA). The results were presented as means ± SD of at least three repeats. *p* < 0.05 was considered a statistically significant difference and marked with an asterisk (*) or an octothorpe (#).

## Figures and Tables

**Table 1:** The information for the primary antibodies used in the present work.

Antibody	Cat. No.	Molecular Weight (kDa)	Dilutions
TLR4	sc-293072	95	1:1000
GAPDH	G031	37	1:2000
TRIF	ab139281	76	1:1000
TRAF6	ab137452	60	1:1000
SREBP-2	28212-1-AP	124 (SREBP-2 precursor) and 73 (SREBP-2 mature forms)	1:1000
Lamin B1	12987-1-AP	66	1:1000
MyD88	4283S	33	1:1000
TNF-α	3707S	17	1:1000
NF-κB-p65	4764S	65	1:1000
phospho-NF-κB-p65	3031S	65	1:1000
β-actin	4967S	45	1:1000

## Data Availability

Data are contained within the article and Supplementary Material.

## Supplementary Materials

The following supporting information can be downloaded at: https://www.mdpi.com/article/10.3390/ph15070813/s1. Figure S1: Effect of LPS and PA on TRIF and TRAF6 expression in HepG2 cells. Cells were treated with LPS (1 μg/mL) or PA (200 μmol/L) for 0 min, 10 min, 30 min, 1 h, 3 h, 6 h, 12 h, 24 h, or 48 h. The protein levels of TRIF (A,C) and TRAF6 (B,D) were measured by Western blotting. The results are presented as means ± SD of three independent experiments. *: *p* < 0.05 vs. 0 min; **: *p* < 0.01 vs. 0 min. Figure S2: MyD88 inhibitor ST2825 suppressed the activation of NF-κB in LPS- or PA-treated HepG2 cells. HepG2 cells were pre-treated with 20 μmol/L ST2825 for 2 h and then treated with LPS (1 μg/mL) for 12 h (A) or PA (200 μmol/L) for 24 h (B). The protein levels of p65 and p-p65 were measured by Western blotting. The results are presented as means ± SD of three independent experiments. *: *p* < 0.05 vs. control; **: *p* < 0.01 vs. control; #: *p* < 0.05 vs. LPS or PA alone; ##: *p* < 0.01 vs. LPS or PA alone.
