# Supplementary figures and images for: Inflammation Induced by Lipopolysaccharide and Palmitic Acid Increases Cholesterol Accumulation via Enhancing Myeloid Differentiation Factor 88 Expression in HepG2 Cells
Source: Pharmaceuticals (Basel). 2022 Jun 30;15(7):813. doi: 10.3390/ph15070813 (PMC9322353; doi:10.3390/ph15070813)

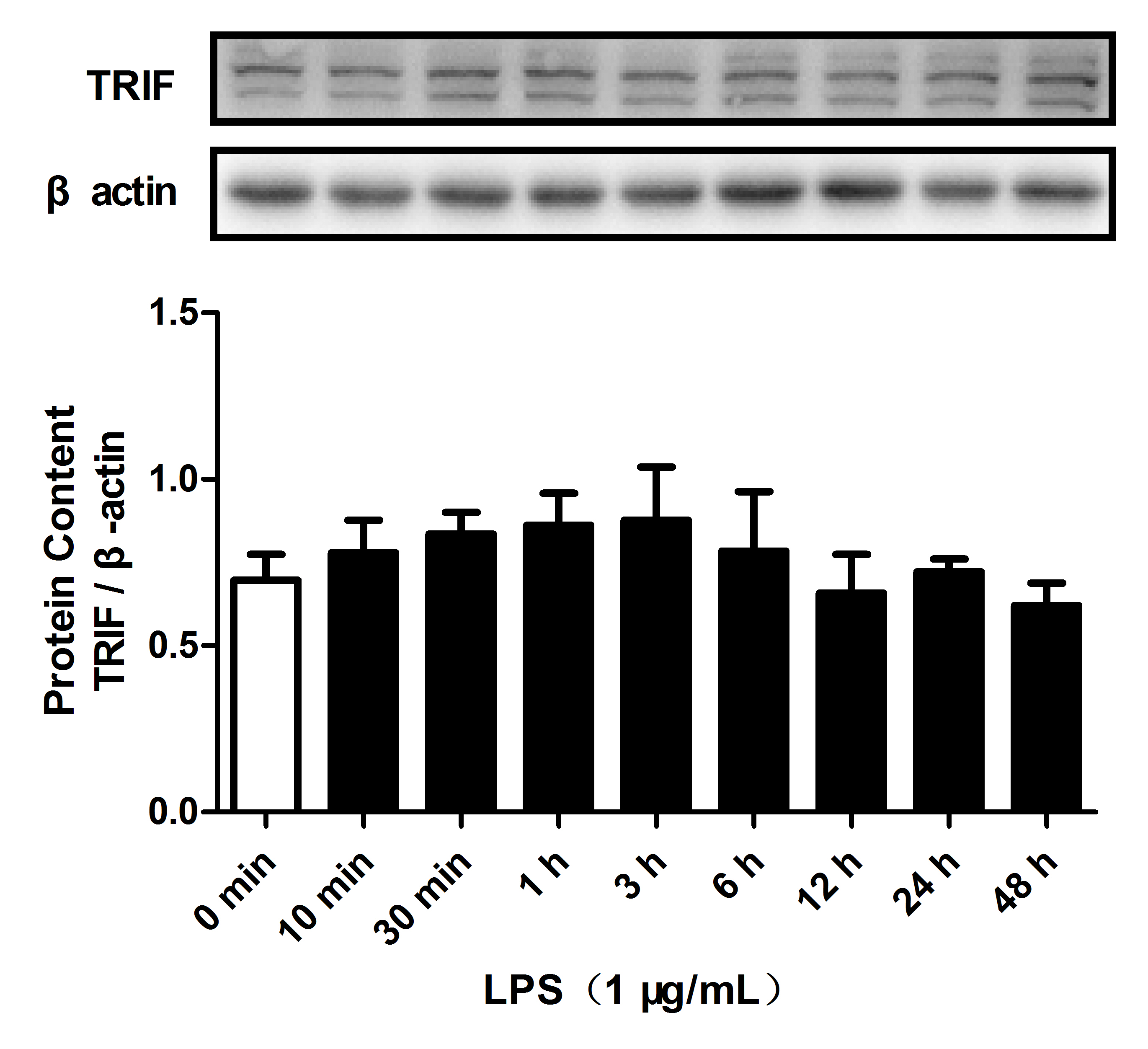

Supplement: Supplementary file 1 [file pharmaceuticals-15-00813-s001.zip › Supplementary files/Figure S1/A.jpg]

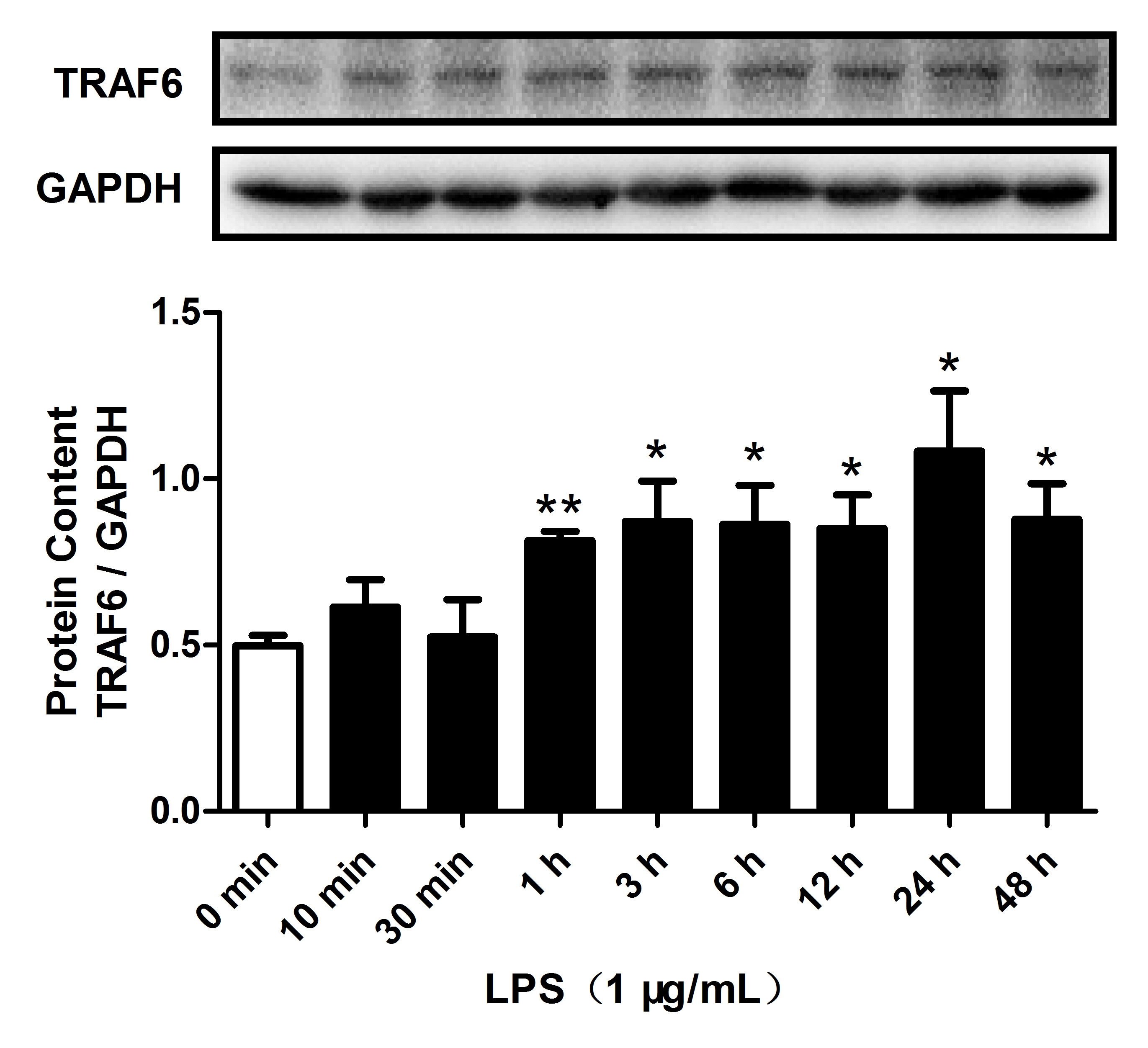

Supplement: Supplementary file 1 [file pharmaceuticals-15-00813-s001.zip › Supplementary files/Figure S1/B.jpg]

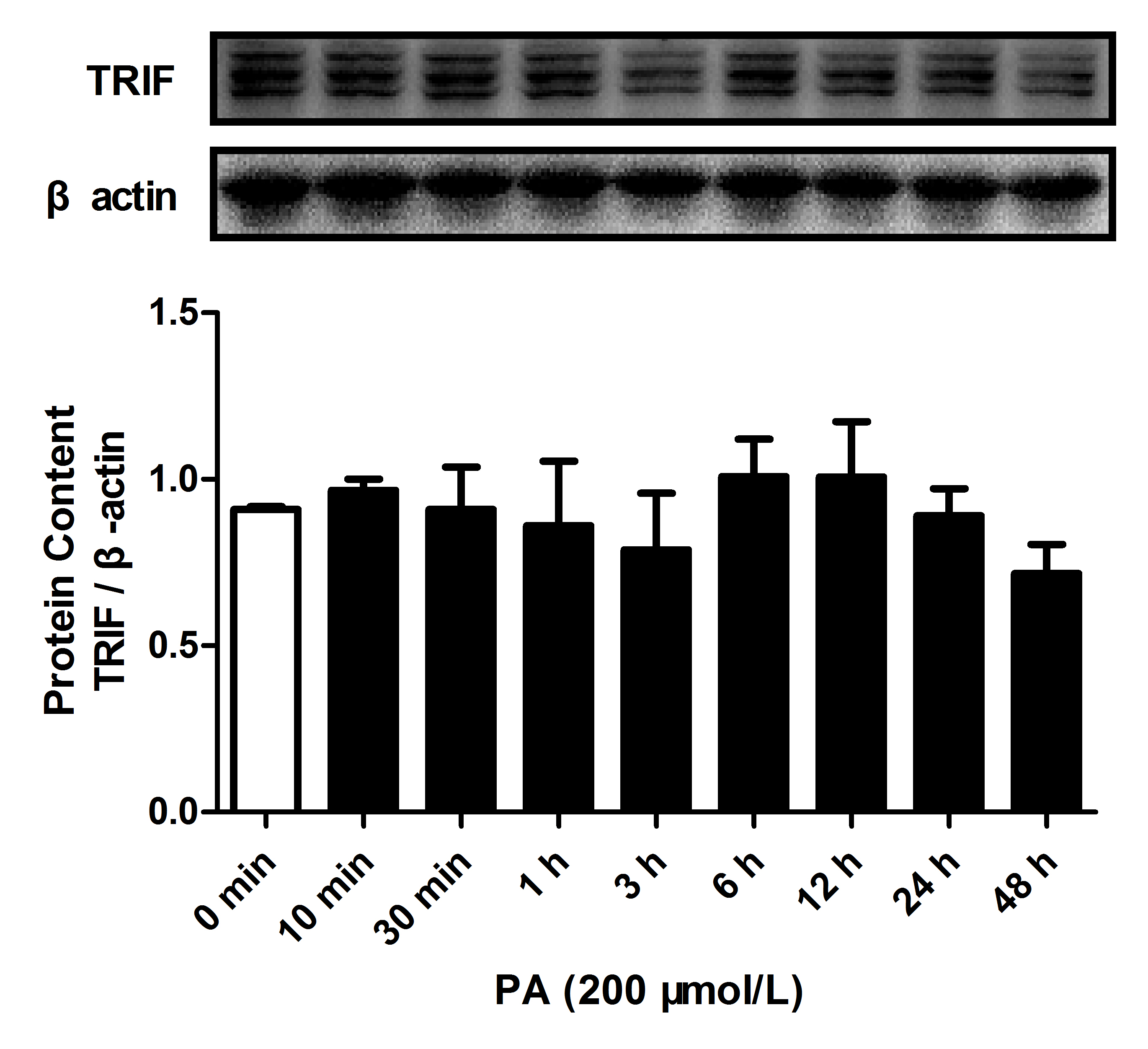

Supplement: Supplementary file 1 [file pharmaceuticals-15-00813-s001.zip › Supplementary files/Figure S1/C.jpg]

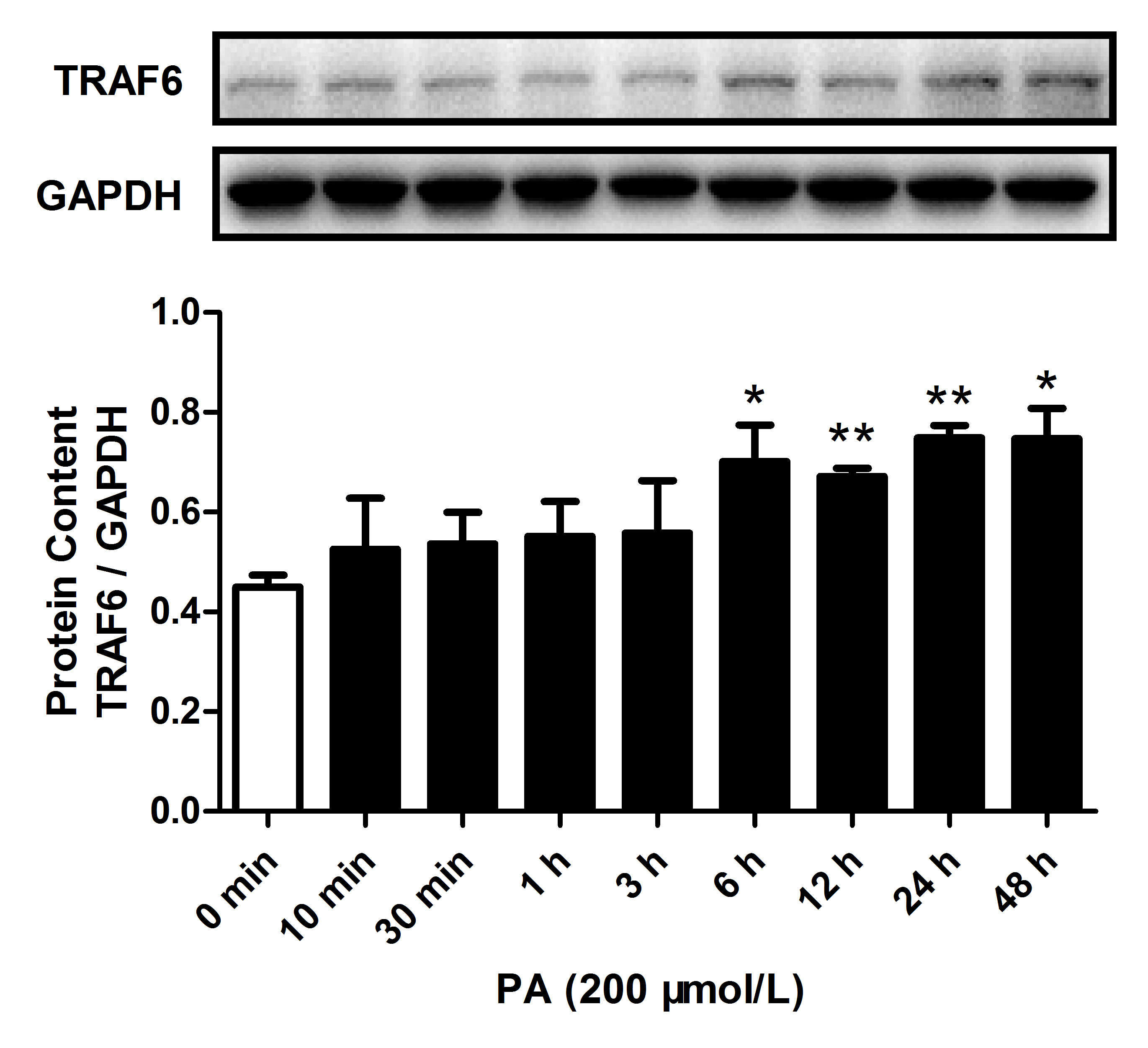

Supplement: Supplementary file 1 [file pharmaceuticals-15-00813-s001.zip › Supplementary files/Figure S1/D.jpg]

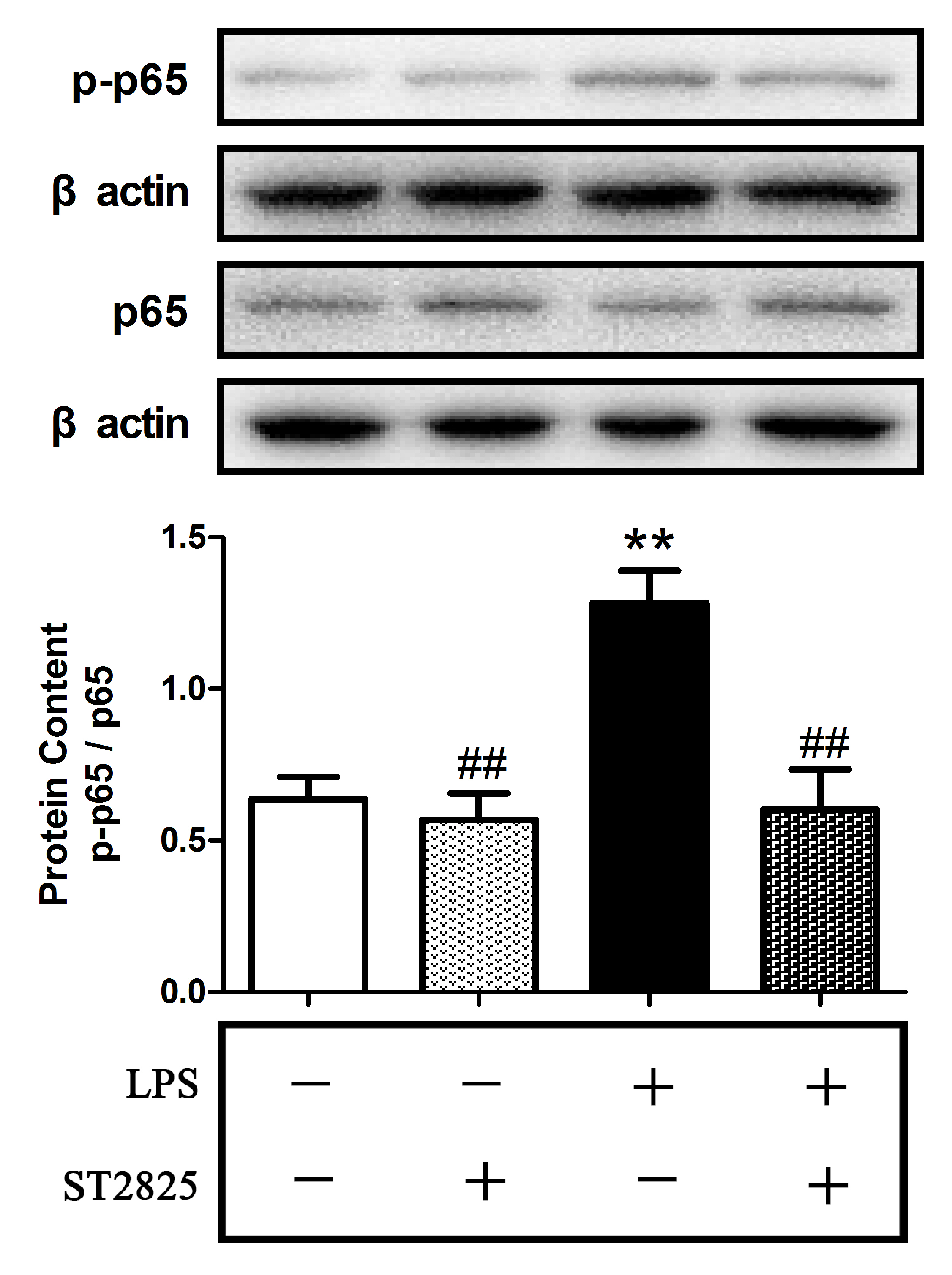

Supplement: Supplementary file 1 [file pharmaceuticals-15-00813-s001.zip › Supplementary files/Figure S2/A.jpg]

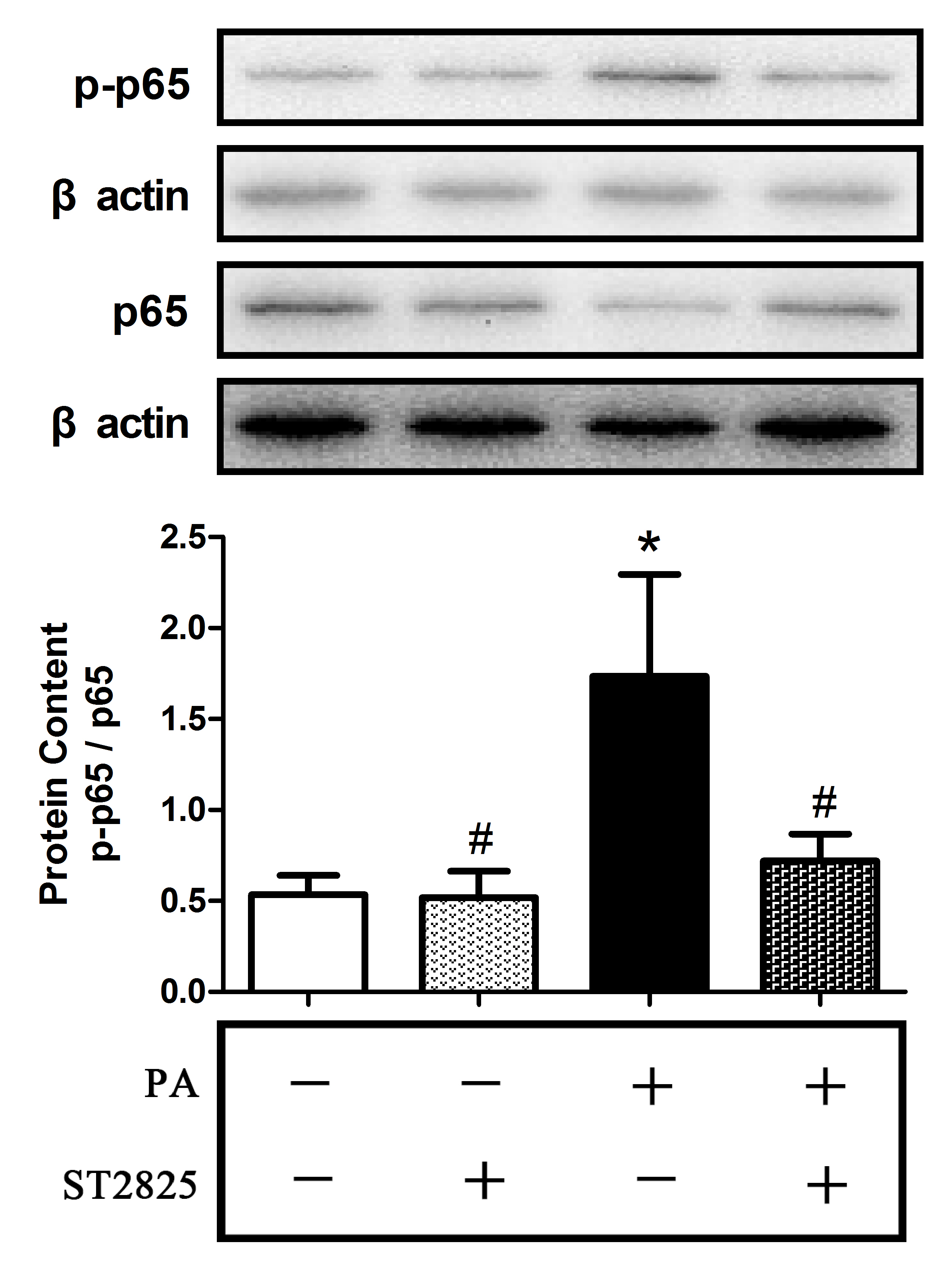

Supplement: Supplementary file 1 [file pharmaceuticals-15-00813-s001.zip › Supplementary files/Figure S2/B.jpg]
